# The dynamics of organ donation in Palestine: legal, religious, and socioeconomic perspectives in a complex political and economic landscape

**DOI:** 10.3389/fpubh.2025.1516865

**Published:** 2025-01-17

**Authors:** Muayad K. Hattab, Belal M. Rahhal, Fayez Mahamid, Noor O. Adas, Rana Dawas, Nidal Jaradat, Hamzeh Al Zabadi, Iyad Ali, Jamal Kielani, Ali Musleh, Ghassan Khaled

**Affiliations:** ^1^Department of Law, Faculty of Law and Political Science, An-Najah National University, Nablus, Palestine; ^2^Department of Biomedical Sciences, Faculty of Medicine and Health Sciences, An-Najah National University, Nablus, Palestine; ^3^Department of Psychology, Faculty of Business and Communication, An-Najah National University, Nablus, Palestine; ^4^Faculty of Medicine and Health Sciences, An-Najah National University, Nablus, Palestine; ^5^Faculty of Sharia, An-Najah National University, Nablus, Palestine

**Keywords:** organ donation, religious beliefs, socioeconomic factors, public awareness, Palestinian healthcare, public attitude, Palestinian legal framework

## Abstract

**Background:**

Organ donation in Palestine is influenced by a unique blend of legal, religious, and socioeconomic factors compounded by the region’s political and economic instability. Despite the increasing need for organ transplantation, public hesitation and misconceptions hinder participation. This study aimed to explore these dynamics, identify barriers, and provide insights into improving organ donation practices in Palestine.

**Methods:**

A quantitative survey was conducted among 639 Palestinian adults residing on the West Bank. The survey assessed participants’ attitudes, awareness, and perceptions of organ donation across three domains: religious/psychological, medical, and legal. The data were analyzed using chi-square tests and three-way ANOVA to explore differences in attitudes based on demographic factors such as sex, academic level, and place of residence.

**Results:**

The study revealed significant variability in public attitudes toward organ donation, with 43.4% willing to donate organs after death but 30% still opposed. Religious and psychological awareness significantly influenced participants’ attitudes, with 40.35% agreeing that organ donation aligns with their beliefs. Medical awareness was higher, with 48.8% feeling informed about the process. Legal awareness was moderate, with 44.85% understanding of the legal framework. The analysis showed no significant demographic differences in attitudes toward organ donation.

**Conclusion:**

Public awareness, religious beliefs, and socioeconomic conditions play crucial roles in shaping organ donation attitudes in Palestine. This study highlights the need for targeted educational campaigns that integrate religious perspectives and raise awareness about the legal and medical aspects of organ donation. Strengthening legal frameworks and healthcare infrastructure will be key to improving organ donation rates and ensuring ethical practices in the region.

## 1 Introduction

The dynamics of organ donation encompass a broad spectrum of studies ranging from public awareness and ethical considerations to policy efficacy and medical outcomes. Organ transplantation has significantly advanced, demonstrating the feasibility of replacing damaged organs and substantially improving quality of life and survival rates. However, the primary challenge in organ transplantation programs, particularly in Middle Eastern countries, is donor organ shortages, which constrain transplant opportunities (1). As a result, high public awareness and positive attitudes toward organ donation play crucial roles in maintaining and enhancing the effectiveness of these life-saving medical procedures (2). Ensuring a supportive public environment for organ donation also relies heavily on a reliable and efficient legal system supported by functional medical practices (3).

In the landscape of organ donation within Palestine, the interplay of ethical, legal, and socioeconomic dimensions creates a complex framework that warrants careful examination. This dynamic is further complicated by the region’s unique political and social context, where poverty, coercion, and cultural factors intersect, influencing individuals’ decisions and perspectives on organ donation and selling (4). The complexity of political adversity is not limited to ongoing occupation in Palestinian territories; it is also exacerbated by travel restrictions and internal divisions of authority between the Gaza Strip and the West Bank (5). These factors contribute to limitations in organ donation and significant economic hardship for a substantial segment of the population (6). Moreover, legislative shortcomings, medical ethics, and public health policies add further layers of complexity, complicating efforts to create a vigorous legal framework that effectively supports organ donation.

The public perception of organ donation varies significantly within Palestinian society and is influenced by cultural, religious, and ethical considerations. These factors necessitate a nuanced approach to understanding and addressing diverse views and concerns. Misconceptions and religious misinterpretations often lead to hesitancy in organ donation, which underscores the need for targeted awareness campaigns that align with societal values and religion (7).

Given these dynamics, Palestine—with its unique economic, political, and cultural landscape—is a compelling case study for understanding the evolution of organ donation practices. This article delves into a comprehensive survey conducted in Palestine aiming to unravel the intricacies of public acceptability and the underlying psychology and motivations of the Palestinian community toward organ donation. By shifting the focus from mere motivation to the broader concept of acceptability, the study seeks to capture the societal impact of organ donation, ensuring the long-term viability of organ transplantation as a respected medical intervention. This article sheds light on the vital interplay between individual autonomy, policy-making, and societal endorsement of organ donation, framing a nuanced understanding essential for nurturing and sustaining public support in this complex environment.

## 2 The framework of organ donation in Palestine

Organ donation is a critical medical practice that has the potential to save lives and improve the quality of life for countless individuals (8). However, its complexities span legal, ethical, religious, and socioeconomic realms (9). Understanding these dimensions is key to developing effective organ donation systems, especially in regions with unique cultural, political, and economic contexts like Palestine.

This review examines the historical and legal framework, ethical standards, religious and cultural perspectives, and socioeconomic factors that shape organ donation in Palestine, highlighting the challenges and opportunities in this essential healthcare sector.

### 2.1 Historical and legal context

Until 2017, Palestine’s legal framework for organ transplantation was underdeveloped. The Mecelle (Journal of Judicial Rules), based on classical Islamic jurisprudence, provided a broad regulatory backdrop, but it lacked specific provisions for organ transplantation (10). The Penal Code No. (16) of 1960 partially addressed this gap. Article 89’s necessity clause allowed organ transfers under certain conditions, weighing potential harm against benefits (11). Similarly, Article 62/c permitted surgical operations with consent or in cases of necessity, indirectly covering organ transplantation. Early legislation like the Law for Utilizing the Deceased’s Eyes for Medical Purposes, No. (43) of 1956, focused solely on using corneas from deceased donors (12). However, more comprehensive laws were needed to address broader organ donation practices. Most countries have enacted laws to regulate organ donation centers, protect donor and patient rights, and prevent exploitation. The right to life and access to medical treatment, as affirmed by international law and Palestine’s Basic Law, are key considerations (13). The Public Health Law No. 20 of 2004 (14) established general controls for medical procedures, including organ transplantation, though without detailed regulations (15).

A pivotal moment came in 2013 when the Mufti of Jerusalem and Palestine issued a religious edict permitting organ transplantation under specific conditions, setting the stage for future legal reforms (16). In 2017, Decree-Law No. (6) on Regulating the Transfer and Transplantation of Human Organs explicitly set the conditions for organ transplantation and prohibited organ trafficking (17). Despite these legal advancements, cultural and social resistance continues to limit the law’s effectiveness, highlighting the need for greater public awareness. Further legislation, such as Decree Law No. (31) of 2018 on medical and health protection, provided broader guidelines for surgical procedures and patient safety (18).

The evolution of legal reforms concerning organ transfer and transplantation in Palestine reflects a complex interplay of legal, medical, and cultural factors. The transition from a loosely regulated environment to a more structured legal framework represents a critical step toward integrating Palestine into the global medical community, with organ transplantation as a key focus area. However, the full implementation of these laws is still hindered by the absence of necessary bylaws, supportive rules, and modern ethical codes. These deficiencies continue to strain the successful execution and expansion of these life-saving medical procedures.

### 2.2 Legal and ethical standards and healthcare system

Organ donation is globally recognized as a critical intervention for saving lives and enhancing the quality of life for patients with end-stage organ failure. The World Health Organization underscores the importance of organ donation as a life-saving procedure; however, many countries, including those in the Middle East, such as Palestine, face significant challenges in meeting the demand for organ transplants. These challenges are not limited by a shortage of donors but also by the lack of comprehensive legal and ethical strategies needed to raise public awareness, improve healthcare infrastructure, and address prevalent public misconceptions (7).

The existing legal landscape, as explored by various scholars (19), highlights the urgent need for robust regulations that protect against exploitation while promoting ethical organ donation practices. Alghamdi et al. (20) noted that legal frameworks and ethical standards are essential components in fostering organ donation. This underscores the necessity for comprehensive strategies to increase organ donation rates in a sound legal and ethical context. In Palestine, explicit consent—either from the donor prior to death or from their family afterward—is required for organ retrieval. The Decree of RTTHO ensures these practices meet legal and ethical standards, tackling issues like organ trafficking while respecting donor autonomy and cultural values.

The adoption of brain death criteria, despite initial resistance, reflects the importance of aligning medical practices with legal and ethical norms (21). However, the absence of legislation facilitating organ donation, such as supporting paired organ donations, hinders the expansion of organ transplant options (20). This legislative gap contributes significantly to the low organ donation rate in Palestine.

Furthermore, the infrastructure and policies of the healthcare system are equally critical in supporting organ donation (22). In Palestine, the capacity of the healthcare system to support organ donation—from public awareness campaigns to transplantation and postoperative care—requires careful examination. There is a significant gap in related research regarding the efficacy of these systems and how they can be optimized to significantly influence the success of organ donation programs.

Healthcare providers are key in shaping public attitudes and decision-making around organ donation. Studies show that medical professionals can significantly influence public perceptions and behaviors (20). Thus, developing supportive policies and legal frameworks is essential to improving donation rates. Comparisons of consent models, such as presumed versus explicit consent, highlight their varying impacts on donation rates (23). Vanholder et al. (2) suggest that the absence of supportive legislation partly explains Palestine’s low donation rates. Research by Abdulrazeq et al. (7) further indicates that strong laws promote ethical organ donation practices. Overall, the lack of legislation and regulatory frameworks, particularly in Palestine’s politically and economically complex environment, significantly limits the growth of organ donation options.

### 2.3 Religious and cultural perspectives

Cultural and religious beliefs strongly influence attitudes toward organ donation, especially in Palestine. In Jordan, Aboghazleh et al. (24) found that factors like age, sex, and employment status significantly shape public views on organ donation. Similarly, in Palestine, cultural and religious beliefs either facilitate or hinder donation (25). Misconceptions and religious misinterpretations often cause hesitancy, revealing the complex interplay between religious teachings, cultural norms, and individual beliefs (26). Ethical concerns about selling organs, rather than donating them altruistically, further complicate the issue in these contexts (27).

The role of religion in shaping attitudes toward organ donation varies across different regions. In many European and Western countries, religious identity has minimal impact on attitudes (28, 29). However, in Muslim communities, concerns about religious prohibitions are more pronounced (30). This is particularly true in predominantly Muslim countries, where religion significantly influences both personal and public views on organ donation (31). Research suggests that increased religious education could positively influence donation rates by aligning religious beliefs with medical practices (32).

Debates around organ transplantation within Islamic jurisprudence reflect its modernity, with no explicit guidance found in traditional Muslim texts (33). Historically, orthodox Islamic positions prohibited both organ donation and reception, citing the need to maintain the body’s integrity and the belief that God owns the human body (34). Additionally, concerns about unethical practices, such as organ trafficking, particularly in regions with weak regulations, have fueled opposition (35). However, many argue that these risks can be mitigated through strong ethical guidelines and legal enforcement (36).

In contrast, contemporary Islamic scholars support organ donation, viewing it as a medical advancement that aligns with Islamic values, particularly when saving lives (33). These scholars maintain that Islamic law permits organ donation when it does not endanger the donor’s life and strictly prohibits the sale of organs. This more permissive interpretation is increasingly accepted in the Muslim world as awareness of transplantation’s benefits grows (34).

In Palestine, religious perspectives heavily influence the legal and ethical framework surrounding organ donation (37). Since Islamic scripture lacks explicit prohibitions, the Palestinian Higher Fatwa Council issued a fatwa permitting organ donation under certain conditions (33). Influenced by scholars in Egypt and Saudi Arabia, the fatwa allows organ transfers from both living and deceased donors when it aims to save lives (16). The 2013 fatwa further formalized this stance, outlining specific conditions to ensure ethical compliance.

The fatwa identifies two scenarios: living and deceased donors. For living donors, donation is allowed only if it does not harm essential bodily functions or cause disfigurement. Concerns about genetic lineage have also led to caution in transferring organs with genetic traits. For deceased donors, organ transfer is permitted if ethical guidelines are strictly followed and the donor’s death is confirmed. These religious and ethical considerations are integral to Palestine’s legal framework on organ transplantation, merging religious doctrine with modern medical ethics.

The distressing choices faced by potential organ donors who consider selling their organs to alleviate their own or their families’ financial needs encapsulate a broader ethical dilemma where poverty acts as a coercive force, undermining the autonomy of individuals in making life-altering decisions (38). The literature frequently portrays poverty as a key driver in the organ trade, with impoverished individuals often viewing the sale of organs as a last resort to escape financial destitution (39). This coercive power of poverty challenges the very notion of free will in the decision-making process, suggesting that decisions made under such dire circumstances are inherently compromised.

Legal discussions focus on whether to regulate or prohibit organ sales to prevent exploitation (40). However, the effectiveness of these measures is debated due to the ongoing global demand for organs and disparities in wealth and health (39). The debate around financial incentives versus compensation for donation-related expenses also reveals conflicting public attitudes toward commodifying body parts (41). The fine line between encouraging donation and maintaining ethical integrity highlights the moral complexity of the issue.

In Palestine, stringent laws exist to combat illegal organ trading, but economic and political challenges complicate enforcement. Epstein (42) questioned whether current bioethical frameworks adequately address the realities of individuals choosing between economic survival and bodily integrity. For instance, Palestinian prisoners in Israel who sought permission to sell their kidneys to support their families exemplify how poverty coerces people into making desperate decisions. This lack of legal clarity, combined with economic desperation, fosters conditions ripe for unethical organ trade.

While economic hardship and poverty create a vulnerable segment of the population that may consider selling organs as a viable financial option, some researchers (22) (41) (43) have suggested that the lack of stringent legislation and effective enforcement mechanisms also play a crucial role in perpetuating the trade. The organ market thrives where legal ambiguities or voids allow brokers and other participants to operate with relative impunity. However, other researchers (44) (45) argue that even with strict legal measures taken to combat organ trading, the challenge remains significant, as the effectiveness of such measures is often hindered by the secretive operations and ingenuity of brokers who exploit legal loopholes. These researchers support the view that allowing individuals the option to sell or trade their organs might curb illegal trading by eliminating the pressure and coercion that drive individuals to make these decisions (44). However, this approach raises significant legal concerns about the validity of consent when individuals are driven by economic stress and poverty, given their potentially vulnerable state.

Further research is needed in politically and economically complex regions like Palestine, where the link between organ donation and challenging social conditions remains underexplored. This calls for a broader ethical and legal discussion that respects individual autonomy while addressing the systemic injustices that lead to such difficult choices.

The tension between protecting individual rights and addressing economic exploitation in the organ trade is a persistent theme (46). Discussions around regulating or prohibiting organ sales continue to focus on preventing exploitation (45, 47), but the effectiveness of such measures remains uncertain given the global demand for organs. Whether stricter penalties or broader social justice initiatives are the answer is still a matter of debate.

### 2.4 Public attitudes and awareness

Public perception and knowledge about organ donation in Palestine are crucial for understanding broader societal acceptance and participation in organ donation programs. Research indicates significant variability in public awareness and attitudes, often shaped by misconceptions, cultural beliefs, and the level of trust in medical institutions (20). Enhancing public education and addressing these misconceptions are vital steps toward improving organ donation rates and ensuring ethical practices.

Early research on organ donation frequently focused on donor motivation, revealing the critical role that public perception plays in facilitating organ donation (48). However, a notable gap between positive attitudes and actual donor behavior has often emerged, highlighting the complex interplay between knowledge, ethical considerations, and decision-making processes in organ donation (49). More recent literature, such as the work of Van Dellen et al. (50) and Timar et al. (51), has shifted the focus toward the concept of public acceptability, suggesting that effective organ donation policies must resonate with societal values and norms to ensure sustainability. This juxtaposition of motivational factors with public acceptability represents a paradigm shift, acknowledging that understanding public sentiment involves more than just the willingness to donate; it encompasses broader societal and ethical dimensions (47, 52).

Nordfalk et al. (28) propose that focusing on public acceptability rather than solely on donor motivation is essential for sustaining support for organ transplantation. This perspective emphasizes the importance of aligning organ donation policies with public values and priorities to create sustainable medical practices. Similarly, Tontus (53) reported that increasing the number of organ donors is critical for extending the benefits of transplantation to a larger patient pool. Factors such as age, sex, socioeconomic status, and education significantly shape attitudes toward organ donation. Additionally, cultural beliefs, ethical considerations, and religious views play influential roles in forming these attitudes. Projects aimed at raising awareness about organ donation, particularly those that seek to transform knowledge into action, are deemed essential. Integrating organ donation-focused education across all levels of schooling is considered a vital strategy for fostering positive attitudes and increasing donation rates (20).

Moreover, awareness and knowledge about organ donation are pivotal in shaping public attitudes. Despite a generally positive disposition toward organ donation, there is still a significant lack of awareness about the process and the legalities involved (24) (31). This gap underscores the need for targeted educational campaigns to improve the understanding and support of organ donation. In the Palestinian territories, similar initiatives could effectively address prevalent myths, increase awareness, and promote a more positive attitude toward organ donation.

The debate over consent models further exemplifies the critical role of public opinion in shaping organ donation policies. While presumed consent policies have been successful in some regions, people often encounter public resistance due to concerns about autonomy and ethical implications (54). Studies such as those conducted by Irving (26) and Abdulrazeq et al. (7) have explored the multifaceted nature of organ donation, emphasizing the significant role that public awareness and cultural perceptions play. Irving ‘s (26) research highlighted how societal norms and knowledge levels significantly affect organ donation decisions, a theme echoed in the Jordan-based study by Abdulrazeq et al. (7), which identified similar barriers related to knowledge and attitudes. Nordfalk et al. (28) suggest that the public’s preference for informed consent and mandatory registration reflects a societal inclination toward maintaining individual control and decision-making authority in the donation process.

## 3 Methods

A quantitative approach was employed for the purpose of the study. This approach focuses on collecting numerical data by using a survey tool. Participants were informed about the nature and procedure of the investigation and gave electronic consent before completing the questionnaires. Participation was voluntary. With this approach, we investigated the complex and multifaceted nature of organ donation in Palestine.

### 3.1 Participants

The study sample consisted of 639 Palestinian adults residing on the West Bank of Palestine. Participants were aged 18 years and older, with an almost equal gender distribution. The study tool was disseminated through social media and official platforms, including the An-Najah National University website—the largest university in Palestine, with approximately 26,000 students from various disciplines—ensuring accessibility to both students and academic staff (55). Additionally, due to the unique circumstances in Palestine, the sample included individuals living in refugee camps, cities, and villages, ensuring representation across diverse cultural backgrounds and residential settings within the region.

### 3.2 Measures

The primary instrument used in this study was a Personal Information Questionnaire designed to collect demographic data such as academic level, sex, age, and place of residence. The questionnaire also included items assessing participants’ attitudes and perceptions toward organ donation. It was administered online and consisted of structured interview questions, with no open-ended questions included. The questions were predetermined in both topic and order, ensuring consistency across all participants. Responses were collected using a 3-point Likert scale, which was subsequently coded and analyzed.

### 3.3 Translation process

The researchers employed a conceptual equivalence translation method combined with a back translation approach. Initially, the survey items were translated from Arabic to English by a first translator. The researchers then thoroughly reviewed these translations, identifying and correcting any errors or inaccuracies, particularly those related to technical and psychological concepts.

To ensure translation accuracy, a back translation was performed. The English version was given to a second translator, who had no prior knowledge of the original Arabic content, to translate it back into Arabic. The researchers then compared the back-translated text with the original Arabic content, focusing on maintaining conceptual equivalence. This process revealed 93% agreement between the translated and original versions. Given the high level of consistency and the constraints of time, the decision was made not to involve a panel of translation experts.

### 3.4 Procedures

The research was conducted from November 2023 to April 2024, targeting Palestinian adults on the West Bank. The study received ethical approval from the Institutional Review Board (IRB) of An-Najah National University prior to data collection. Participants were recruited online, which allowed for broader reach but may have excluded individuals without internet access. The aims and procedures of the study were clearly explained to participants online, including issues of confidentiality and the voluntary nature of participation. Those who agreed to participate provided informed consent and subsequently provided access to the study instruments online. The completed questionnaires were returned electronically and analyzed by the research team.

### 3.5 Data analysis

To examine the significance of differences in participants’ awareness and attitudes, a chi-square test was performed. Furthermore, a three-way ANOVA was conducted to assess the influence of demographic factors—gender, academic level, and place of residence—on attitudes toward organ donation.

## 4 Results and findings

The results of the study provide a comprehensive view of Palestinian adults’ attitudes toward organ donation, reflecting a complex interplay of religious beliefs, psychological awareness, medical understanding, and legal awareness. The survey revealed that Palestinian adults exhibit a generally positive attitude toward organ donation, although there are significant variations depending on the specific context. For instance, 43.4% of participants agreed with organ donation under certain conditions, 26.6% remained undecided, and 30% disagreed, as presented in Table 1.

**Table 1:** Participants’ agreement on organ donation (*n* = 638).

No.	Statement	Agree %	Undecided %	Disagree %	Total %
1	I am willing to donate my organs after death.	43.4	26.6	30	100
2	I am willing to donate only part of my organs after death.	49.2	25.2	25.6	100
3	I am willing to donate a portion of my organs during my lifetime.	21.5	29.7	48.7	100
4	I am willing to donate the organs of my relatives after their death, if that is within my powers.	28.5	26.3	45.3	100
5	I am willing to receive organs from a deceased donor.	50	29.7	20.3	100
6	I am willing to receive organs from a living donor.	33	32.4	34.6	100
7	As a living donor, I am willing to donate my kidney to someone in need.	46.7	30.7	22.6	100
8	I am willing to receive a kidney only from a living donor.	38.5	37.9	23.6	100
9	I believe that community awareness should be increased to encourage more organ donations in my country.	65.9	25.3	8.8	100
10	I will agree to donate an organ only if one of my relatives is in need.	44	30.7	25.3	100
11	I have experienced a relative donating his organ to someone else.	9.4	11.9	78.6	100
12	I have previously donated one of my organs to someone else.	1.9	9.9	88.2	100
13	I have no objection to my family donating my organs after my death.	53.1	21.5	25.3	100
14	I believe that organ donation is prohibited by my religion.	12.4	37.7	49.8	100
15	I dislike thinking about organ donation because it reminds me of death.	21.5	23.1	55.3	100
16	I accept the organ donation between individuals of different religions.	42.5	29.6	28	100
17	I believe that organ donation is a moral obligation.	41.7	39.3	19	100
18	I can forgive myself if my refusal to donate an organ leads to someone’s death.	25.6	38.8	35.5	100
19	I view individuals who refuse to donate organs as lacking generosity.	10.2	34.1	55.7	100
20	Organ donation should be a noble act without financial incentives.	65.1	22.8	12.1	100
21	I appreciate the thought of my body helping save lives after my death.	68.9	19.5	11.6	100
22	I believe it is important for my body to remain untouched after death	33.8	33.8	32.4	100
23	I fear not being fully deceased when doctors proceed with the transplant.	27.5	26.3	46.2	100
24	I am concerned about the potential misuse of donated organs, such as trafficking or medical malpractice.	65.3	20	14.8	100
25	I believe organ donation should occur exclusively at state-licensed centers.	88.7	7.2	4.1	100
26	Medically, an organ donor should be at least 60 years old.	11.6	39.3	49.1	100
27	I believe it is necessary to remove an organ from a deceased individual, even if they had previously refused organ donation.	8.2	14.2	77.7	100
28	Health authorities should facilitate communication between the relatives of the deceased donor and the organ recipient, if requested by both parties.	70.6	20	9.4	100
29	I believe in the possibility of consenting to organ donation from minors or individuals with special needs during their lifetime, with family approval.	11	20.6	68.4	100
30	Similarly, organ donation from minors or individuals with special needs after their death should be possible, with family consent.	45.6	28.1	26.3	100
31	It is crucial to have laws regulating the organ transplant process.	88.5	9.1	2.4	100
32	The organ transplant law is not deemed important or necessary now or in the future.	5	13.5	81.4	100
33	The law should assume consent for organ donation from any deceased person, unless they have explicitly opted out.	17.5	21.4	61.2	100
34	It should be mandatory for individuals over 18 to declare their organ donation preferences and to register these decisions.	50	25.6	24.4	100
35	Incentivizing organ donation through financial compensation should be allowed for donors or their relatives.	17.1	32.2	50.6	100
36	It would be just for donors or their relatives to receive compensation for potential donation-related expenses.	42.1	32.1	25.8	100
37	The state must do more to foster and promote organ donation.	57.7	30.8	11.5	100
38	Laws must protect the rights and safety of both living and deceased donors to encourage organ donation.	79.2	15.3	5.5	100
39	I am fully aware of the legal stance on organ transplantation.	18.6	40.9	40.6	100
40	I understand that the law permits the donation of any organ without exception.	15.3	34.1	50.6	100
41	I am aware that the law prohibits financial transactions for organ donation.	37.4	40.9	21.7	100
42	I am informed that the law criminalizes organ trafficking.	73.7	16.4	9.9	100
43	I believe the law mandates obtaining informed consent prior to organ donation.	81	15.3	3.8	100
First domain		40.35	28.02	31.6	100
Second domain		48.08	21.96	29.93	100
Third domain		44.85	25.2	29.95	100
Total score		43.15	26.4	30.79	100

A key finding of the study was the influence of religious and psychological awareness on attitudes toward organ donation. The data showed that 40.35% of participants agreed with statements relating to the compatibility of organ donation with religious beliefs and psychological well-being, while 31.6% disagreed, as shown in Table 1.

Furthermore, the participants displayed a relatively high level of medical awareness regarding organ donation; 48.8% agreed that they had sufficient medical knowledge about the process, while 29.93% disagreed, as presented in Table 2. Legal awareness emerged as another critical factor influencing attitudes toward organ donation. Approximately 44.85% of participants agreed that they understood the legal framework governing organ donation in Palestine, whereas 29.95% disagreed, as shown in Table 1.

**Table 2:** Results of the chi-square test for differences in organ donation (*n* = 638).

Organ donation domain	Category	Observed N	Expected N	Residual	χ^2^ value	df	*p*-value
Religious and Psychological Awareness	Disagree	202	212.7	−10.7	15.10	2	<0.001
	Undecided	179	212.7	−33.7			
	Agree	257	212.7	44.3			
Medical Awareness	Disagree	191	212.7	−21.7	68.88	2	<0.001
	Undecided	140	212.7	−72.7			
	Agree	307	212.7	94.3			
Legal Awareness	Disagree	191	212.7	−21.7	40.04	2	<0.001
	Undecided	161	212.7	−51.7			
	Agree	286	212.7	73.3			
Total Awareness	Disagree	195	212.7	−17.7	29.11	2	<0.001
	Undecided	168	212.7	−44.7			
	Agree	275	212.7	62.3			

The results indicated significant differences across the three domains of awareness—religious/psychological, medical, and legal—with all three showing *p* values less than 0.001. This statistically significant variation suggests that different segments of the population are influenced by varying factors in regard to organ donation. The analysis revealed no significant differences based on these demographic variables, indicating that attitudes toward organ donation in Palestine are relatively consistent across different population groups.

**Figure 1:**
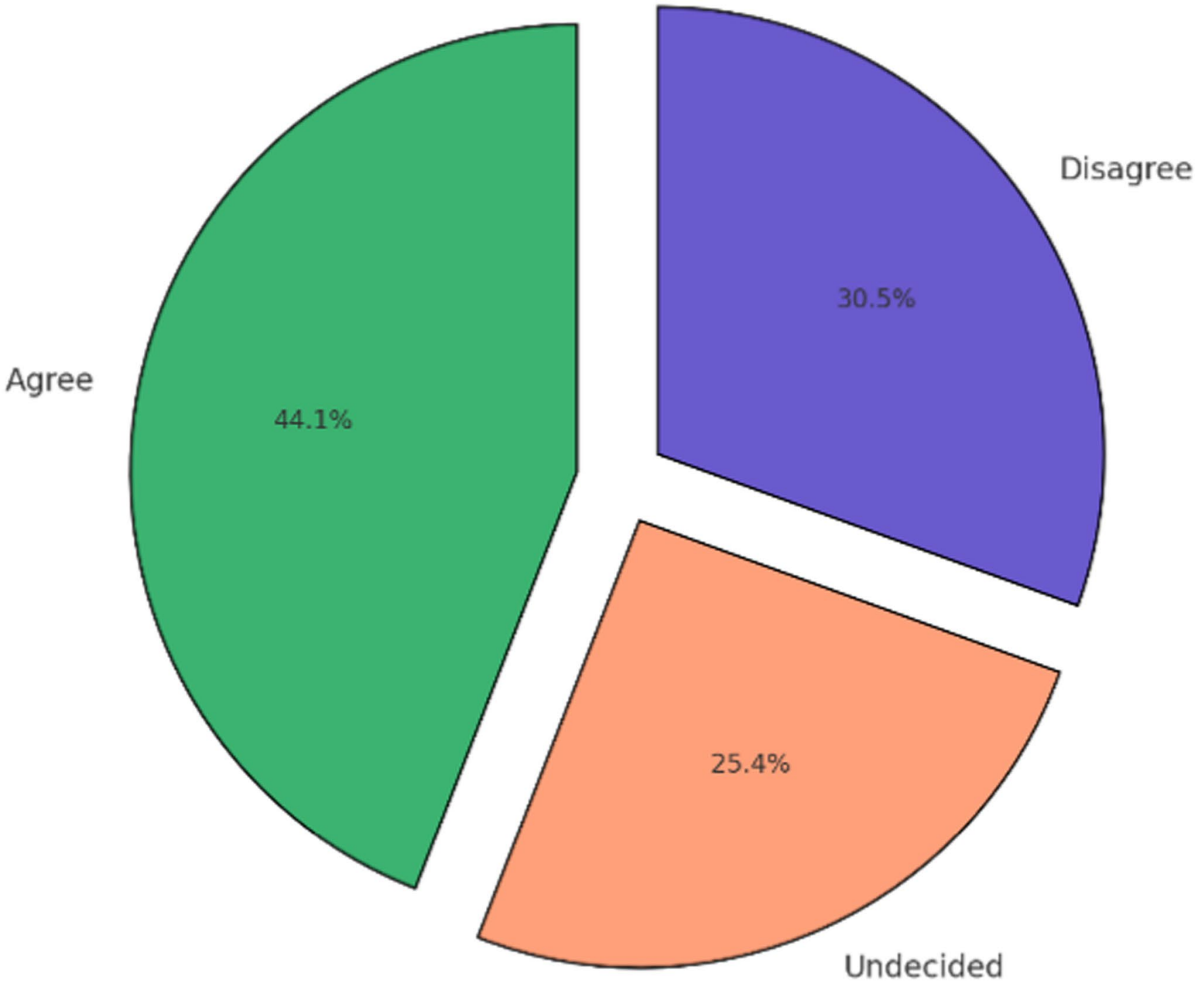
Overall participants’ agreement with organ donation.

**Figure 2:**
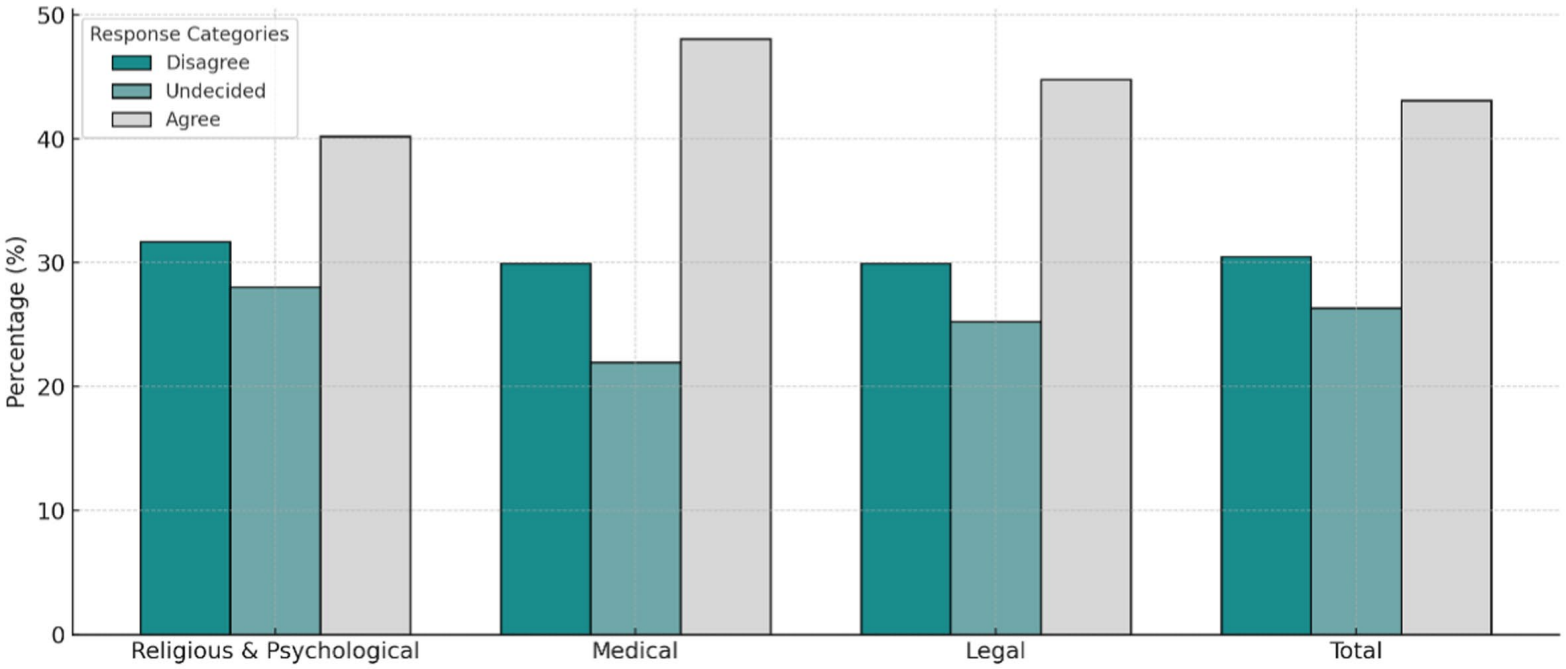
Agreement across different domains.

The results from Table 1 and Figures 1, 2 demonstrate various insights into participants’ opinions about organ donation:

Figure 1 provides a summary of the participants’ overall agreement with organ donation. Most participants had a positive opinion, with 44.1% agreeing, 25.4% undecided, and 30.5% disagreeing. This high level of agreement suggests a generally favorable perception of organ donation among the respondents.

Figure 2 delves deeper into the distribution of agreement, undecided, and disagreement levels across different domains. Religious and psychological awareness, 40.2% of participants agreed that organ donation is compatible with their religious and psychological beliefs, while 31.7% disagreed. Further, in relation to medical awareness, 48.1% of participants indicated sufficient knowledge about the medical aspects of organ donation, while 29.9% disagreed. As to the legal Awareness, 44.8% of participants understood the legal frameworks surrounding organ donation, though 29.9% disagreed.

These figures reflect that while agreement is generally high across domains, there are notable portions of participants who remain undecided or disagree, particularly in the legal awareness domain.

To test the significance of these differences, the chi-square test was performed as shown in Table 2.

Explanation of Terms in Table 2:

- Observed N: The number of participants observed in each category.
- Expected N: The expected number of participants if there were no differences across groups.
- Residual: The difference between Observed N and Expected N.
- χ^2^ value: The chi-square test statistic calculated for each domain.
- df: Degrees of freedom, representing the number of categories minus one.
- *p*-value: The probability of obtaining the observed results under the null hypothesis. A value less than 0.05 indicates statistical significance.

Table 3: Results of the Three-Way ANOVA for Total Score of Organ Donation Awareness (*n* = 638).

**Table 3:** Results of the chi-square test for differences in organ donation (*n* = 638).

Dependent variable	Source	SS	df	MS	F	*p*-value
Organ donation	Gender	0.03	1	0.03	0.07	0.71
	Place	0.01	2	0.006	0.036	0.96
	Academic	0.05	3	0.04	0.09	0.85
	Error	105.19	632	0.175		
	Corrected Total	105.28	638			

A chi-square test was used to assess the significance of the observed differences, as shown in Table 2. The statistics show that people’s emotions differ greatly. The individuals had significant psychological and theological understanding of organ donation (χ^2^ = 15.07, *p* < 0.001). Individuals demonstrated good grasp of organ donation (χ^2^ = 68.88, *p* < 0.001). Participants demonstrated high levels of legal awareness (χ^2^ = 15.07, *p* < 0.001). Participants reported strong overall awareness of organ donation (χ^2^ = 29.11, *p* < 0.001).

Explanation of Terms in Table 3:

- SS (Sum of Squares): Measures the total variation attributable to each factor.
- df (Degrees of Freedom): The number of independent values or quantities that can vary.
- MS (Mean Square): The average of squared differences (SS divided by df).
- F (F-Statistic): A ratio of variance estimates to test the null hypothesis.
- *P*-value: Indicates whether the observed differences are statistically significant. A value greater than 0.05 suggests no significant difference.

To test the significance of differences in the total score for organ donation awareness, a three-way ANOVA was performed (as shown in Table 3). Table 3 shows no significant differences in participants’ awareness of organ donation.

## 5 Discussion

Organ transplantation continues to advance, becoming the preferred treatment for organ failure (56). However, despite global progress in transplant technology, there is still a significant shortage of donor organs, as the demand has not kept pace with advancements (33). In Palestine, the dynamics of organ donation are shaped by a complex interaction of legal, ethical, religious, and socioeconomic factors. While progress has been made in formalizing the legal framework for organ donation, several challenges remain. This discussion interprets the study’s findings within the broader literature, considers implications for policy and practice, and identifies areas for future research.

### 5.1 Legal and ethical challenges

The evolution of Palestine’s legal framework has made significant strides toward formalizing organ donation, as discussed in the section 2. However, the study’s findings indicate that while legal awareness is relatively high, nearly 30% of the population remains unclear or unaware of the legalities surrounding organ donation. This lack of awareness is a substantial barrier to increasing donation rates. Although legal reforms like Decree Law No. (6) of 2017 and Decree Law No. (31) of 2018 are crucial, their effectiveness is limited if the public does not fully understand or trust these regulations.

The gap in legal awareness suggests that enacting laws alone is insufficient. There must be an active effort to educate the public about their rights and obligations under the law. Furthermore, the effectiveness of these laws is weakened by the absence of bylaws, practical guidelines, and resistance from cultural and social factors. To address this, a comprehensive strategy is needed, including public education campaigns, the development of clear legal guidelines, and stronger enforcement of existing laws to build trust and confidence in the organ donation system.

### 5.2 Religious and cultural influences

The study highlights the significant influence of religious beliefs and cultural norms on attitudes toward organ donation. While more than 40% of participants believe organ donation aligns with their religious beliefs, a significant portion remains undecided or opposed. This ambivalence can largely be attributed to misconceptions and religious misinterpretations that persist within the community.

The endorsement of organ donation by the Palestinian Higher Fatwa Council, under specific conditions, is a positive step. However, the ongoing hesitancy among the public suggests more work is needed to reconcile religious beliefs with medical practices. Involving religious leaders in public education efforts could play a crucial role. Religious authorities have the power to clarify religious positions on organ donation and address the community’s ethical concerns. This approach aligns with findings from other Middle Eastern contexts, where religious approval has been shown to significantly influence public acceptance of organ donation.

### 5.3 Socioeconomic factors and ethical dilemmas

The intersection of socioeconomic conditions with organ donation presents complex ethical dilemmas. The study’s findings indicate that poverty plays a significant coercive role, leading individuals to consider selling their organs as a desperate measure. This raises serious concerns about the voluntariness of consent in such cases, as decisions made under economic duress are inherently compromised.

Though Palestinian law prohibits organ trafficking, enforcement is complicated by the region’s economic hardships and political instability. The findings suggest that efforts to curb illegal organ trading will remain insufficient unless the root causes of poverty are addressed. Policies should go beyond legal reforms and tackle broader socioeconomic issues. Economic support programs for vulnerable populations could alleviate the financial pressures that drive people to consider organ selling, ensuring that organ donation remains a voluntary and altruistic act. These conclusions align with previous studies, which found that many individuals are willing to donate organs if someone needs one (57, 58), and that others are willing to donate posthumously (59, 60).

### 5.4 Implications for healthcare systems and policy

These findings also point to significant challenges within the healthcare system that hinder the effective implementation of organ donation programs. While medical awareness is relatively high, a considerable portion of the population still lacks adequate information or has misconceptions about the medical aspects of organ donation. This gap underscores the need for improved public health education, with a focus on increasing the understanding of the medical procedures involved in organ donation and transplantation.

Healthcare providers are uniquely positioned to influence public attitudes and behaviors regarding organ donation. The findings suggest that targeted training for medical professionals on how to discuss organ donation with patients could significantly impact public perceptions and willingness to donate. Additionally, improving the healthcare infrastructure, particularly in areas such as postoperative care and transplantation facilities, is essential for supporting organ donation initiatives. A previous study performed by Altraif et al. (61) revealed that healthcare providers exhibit a more favorable positive attitude toward and possess a greater level of awareness of organ donation in the community (61).

### 5.5 Future research and practical applications

The finding underscores the potential for broad public support for organ donation, provided that educational and policy interventions are appropriately implemented. By concentrating on the shared attitudes across the population, policymakers can design more effective and inclusive strategies to encourage organ donation. The study also highlights several areas where further research is needed. Future studies should explore the impact of specific educational interventions on public attitudes toward organ donation, particularly those involving religious leaders or addressing economic concerns. Additionally, research should investigate the long-term effects of legal reforms on organ donation rates and assess the effectiveness of enforcement mechanisms in preventing illegal organ trafficking. Practical applications of this research include developing targeted public health campaigns that address both religious and economic concerns, creating clear and accessible legal guidelines for organ donation, and implementing economic support programs to reduce the financial pressures that may lead individuals to consider selling organs.

## 6 Conclusion

This study explored the intricate dynamics of organ donation in Palestine, highlighting the significant interplay between legal, ethical, religious, and socioeconomic factors. This research underscores the progress made in formalizing organ donation practices through legal reforms; however, it also identifies persistent challenges related to public awareness, religious beliefs, and socioeconomic conditions. The findings reveal that while there is a general acceptance of organ donation among Palestinians, misconceptions and religious concerns continue to impede broader participation. Furthermore, economic hardships exacerbate the ethical dilemmas associated with organ donation, particularly in terms of coerced consent due to financial pressures.

To address these challenges, a multifaceted approach is necessary. This approach should include enhanced public education initiatives, especially those involving religious leaders, to align religious beliefs with medical practices. Additionally, strengthening the legal framework and improving healthcare infrastructure are crucial steps toward increasing organ donation rates and ensuring ethical practices.

Future research should continue to explore the impact of these interventions on public attitudes and behaviors, with an emphasis on developing strategies that are culturally and contextually relevant to the Palestinian population and those facing similar political or economic challenges. To ensure the success of organ donation and transplantation, further efforts are needed to educate the public, enhance legal and healthcare systems, and address the socioeconomic factors that influence decision-making. These efforts can make meaningful contributions to global organ donation initiatives, ultimately saving lives and improving the quality of life for those in need.

## Data Availability

The original contributions presented in the study are included in the article/Supplementary material, further inquiries can be directed to the corresponding author.
